# Evaluation of the IMPROVE-DD score in COVID-19 patients submitted to venous thromboembolism investigation at a hospital in Brazil

**DOI:** 10.36416/1806-3756/e20240042

**Published:** 2025-03-18

**Authors:** Ronney Argolo Ferreira, Lian Zanatta, Juliane Bispo de Oliveira, Janaina Ibele Carvalho Gomes, Luiz Ritt, Ana Thereza Cavalcanti Rocha

**Affiliations:** 1. Faculdade de Medicina, Universidade Federal da Bahia, Salvador (BA), Brasil.; 2. Escola Baiana de Medicina e Saúde Pública, Salvador (BA), Brasil.; 3. Instituto D’Or de Pesquisa e Ensino, Salvador (BA), Brasil.

**Keywords:** Venous thromboembolism, COVID-19, IMPROVE-DD

## Abstract

**Objectives:**

To evaluate the incidence of venous thromboembolism (VTE) in hospitalized patients with COVID-19 who underwent diagnostic tests for suspected VTE, and to correlate the IMPROVE-DD score with the incidence of VTE in this cohort.

**Methods:**

This retrospective study included consecutive patients with COVID-19 and suspected VTE, admitted between March 2020 and September 2021 at a private hospital in Salvador (BA), Brazil, who underwent lower or upper limb venous Doppler ultrasound or chest angiotomography. Descriptive analyses and comparisons using the chi-square test were performed to identify factors potentially associated with the risk of VTE.

**Results:**

A total of 517 patients were included, with an in-hospital VTE incidence of 18.6% (96 events). Risk factors significantly associated with VTE included obesity, ICU admission, central venous catheter use, longer hospital stays, greater lung tomographic involvement/severity, the need for mechanical ventilation, D-dimer levels at least twice the upper limit of normal (2xULN), and the IMPROVE-DD score. The mean IMPROVE-DD score among patients with VTE was 4.7 (±3) versus 3.3 (±2.4) in those without VTE (p < 0.0001). D-dimer 2xULN was sensitive in identifying 94% of the 96 patients with VTE (p < 0.0001). The in-hospital mortality rate was 14.1%, with higher rates observed in patients with VTE (24%) compared to those without VTE (11.9%) (p = 0.003).

**Conclusions:**

The incidence of VTE in hospitalized COVID-19 patients was high and correlated with increased mortality. The IMPROVE-DD score effectively identified patients at risk for in-hospital VTE, suggesting it could help to identify a high-risk subgroup that may benefit from extended thromboprophylaxis.

## INTRODUCTION

Severe Acute Respiratory Syndrome Coronavirus 2 (SARS-CoV-2) causes Coronavirus Disease 2019 (COVID-19), which may require hospitalization and is associated with an increased risk of venous thromboembolism (VTE). In severe cases, the disease can affect the lower respiratory tract, leading to severe acute respiratory syndrome, acute cardiac injury, increased susceptibility to secondary infections, and coagulation disorders.^1^ ^-^ ^3^

COVID-19 can cause endothelial injury during the process of virus penetration and release, as well as through the release of cytokines, primarily interleukin-6 (IL-6), which exacerbates the process of thromboinflammation.^4^ ^,^ ^5^Severely ill patients may experience reduced mobility, which promotes venous stasis-a key factor in thrombosis. Additionally, increased blood hyperviscosity, evidenced by elevated levels of fibrinogen and factor VIII, has been observed.^5^ Consequently, Virchow’s triad is fulfilled, and the association between COVID-19, microthrombosis, and VTE has been documented in several studies, even in patients receiving thromboprophylaxis.^6^

Given the high risk of developing VTE in hospitalized patients with COVID-19, it has become crucial to determine whether a scoring system could identify patients who would benefit from both therapeutic anticoagulation during hospitalization,^7^ and extended post-discharge thromboprophylaxis.^8^ ^-^ ^10^ The International Medical Prevention Registry on Venous Thromboembolism Risk Assessment Model + D-dimer twice the upper limit of normal (2xULN) (IMPROVE-DD VTE RAM) score^11^ ^,^ ^12^ is an adaptation of the widely validated International Medical Prevention Registry on Venous Thromboembolism Risk Assessment Model (IMPROVE VTE RAM) score.^13^ The incorporation of D-dimer levels into the IMPROVE score, thereby creating the IMPROVE-DD score, was designed to enhance its accuracy in predicting VTE risk.^11^

A study conducted between March and May 2020, involving 4,906 patients from the Northwell Health System health plan, guided the implementation of the IMPROVE-DD score throughout its healthcare network, and indicated the use of rivaroxaban (10 mg orally) or low-molecular-weight heparin (LMWH) enoxaparin (40 mg subcutaneously daily, if creatinine clearance ≥ 15 mL/min) for 30 days in COVID-19 patients with IMPROVE-DD scores ≥ 4. The researchers concluded that the use of the scoring system reduced the risk of major thromboembolic events and death by 46%.^10^

Studies on the utility of the IMPROVE-DD score in COVID-19 patients, however, are limited and mostly confined to the United States. This study investigates the incidence of VTE in hospitalized COVID-19 patients with suspected VTE who underwent diagnostic tests at a hospital in Salvador, Brazil, between March 2020 and September 2021. Using these data, the IMPROVE-DD score was evaluated in this population to identify patients at a higher risk of VTE.

## METHODS

Study design: This retrospective, non-interventional observational study was conducted among hospitalized patients diagnosed with COVID-19 at a private hospital in Salvador, Brazil. The study utilized data from the Clinical Registry (CR) of COVID-19 patients who underwent diagnostic tests for VTE. Suspicion of VTE was based on clinical variables and laboratory findings, such as significant increases in D-dimer levels.

From March 2020 to September 2021, all hospitalized patients at the institution with confirmed COVID-19 via RT-PCR or antigen testing, who underwent lower or upper extremity venous Doppler ultrasound (US) and/or chest computed tomography angiography (CTA), were included. Patients were excluded if COVID-19 was not confirmed or if they had not undergone at least one of the specified diagnostic tests required for cohort selection.

Demographic and biometric data, along with admission diagnoses, were collected. They included systemic arterial hypertension (SAH), diabetes mellitus (DM), coronary artery disease (CAD), history of previous VTE, smoking history, cancer, thrombophilia, obesity - BMI ≥ 30 kg/m^2^ and overweight - BMI ≥ 25 kg/m^2^, lower limb immobilization, patient care units (general ward, intensive care unit [ICU], and step-down unit), D-dimer levels (normal range: ≤ 500 ng/mL, measured using enzyme-linked immunosorbent assay [ELISA]), chest CT and CTA findings, lower or upper extremity venous Doppler ultrasound results, and mechanical ventilation data.

Descriptive data analyses of the study sample were presented as percentages, means (± standard deviations [SD]), and medians (interquartile ranges [IQR]), as appropriate. The percentages of patients with positive, negative, and inconclusive VTE test results and their characteristics were compared, focusing mainly on the IMPROVE-DD score, which comprises eight criteria: 1 - history of previous VTE (+3 points); 2 - known thrombophilia (+2 points); 3 - lower limb paralysis during hospitalization (+2 points); 4 - active cancer (+2 points); 5 - immobilization for seven days or more (+1 point); 6 - ICU admission (+1 point); 7 - age ≥ 60 years (+1 point); 8 - D-dimer levels at least twice the upper limit of normal (+2 points). Patients scoring 0-1 were considered low risk; 2-3, moderate risk; and ≥4, high risk, with potential benefit from extended VTE prophylaxis.^8^ ^,^ ^11^ ^,^ ^12^ ^,^ ^14^

Pearson’s chi-square test or Fisher’s exact test, as appropriate for categorical variables, and Student’s t-test or Wilcoxon’s test for continuous variables, were performed using IBM Statistical Package for the Social Sciences (SPSS 21) software. A Receiver Operating Characteristic (ROC) curve was plotted for IMPROVE-DD scores ≥4, and the Area Under the Curve (AUC), sensitivity, specificity, positive predictive value (PPV), and negative predictive value (NPV) were calculated.

The study protocol was approved by the Research Ethics Committee of the D’Or Research Institute (Process No. 4,383,979).

## RESULTS

A total of 535 patients were evaluated; 18 were excluded due to either lack of confirmed COVID-19 or failure to undergo VTE imaging tests during hospitalization. Among the 517 patients included, 232 (44.87%) were female and 285 (55.13%) were male, with a mean age of 60 years (±17). Of the included patients, 201 (38.88%) were aged < 55 years, 152 (29.4%) were aged 55 to 69 years, and 164 (31.72%) were aged ≥ 70 years.

Among the total of 686 diagnostic tests performed for VTE, 286 were lower limb US, 23 were upper limb US, and 377 were chest CT angiography (CTA) scans. During hospitalization, 96 VTE events (18.6%) were diagnosed. Approximately 76.2% of patients received pharmacological prophylaxis for VTE, and among those hospitalized for more than 48 hours, 95.3% received pharmacological prophylaxis.

The clinical characteristics and comorbidities of patients with COVID-19 and suspected VTE are presented in Table 1. The most frequent risk factors for VTE were overweight and obesity, advanced age, ICU admission, presence of central venous catheters (CVC), mechanical ventilation, recent surgery, and more severe tomographic findings related to COVID-19. Common cardiovascular comorbidities included SAH and DM. D-dimer levels upon admission were higher in patients with VTE, with a mean of 12,021 (± 15,473), compared to those without VTE, whose mean was 3,121 (± 5,132) (p < 0.0001).

**Table 1:** Clinical characteristics and comorbidities of hospitalized patients with COVID-19 submitted to VTE investigation.

Variable All	All (%) 517 (100)	With VTE (%) 96 (18.57)	Without VTE (%) 421 (81.43)	p-value
Women	232 (44.9)	38 (39.6)	194 (46.1)	0.26
Men	285 (55.13)	58 (60.4)	227 (53.9)	
≥ 55 years old	316 (61.1)	64 (66.78)	252 (59.9)	0.47
≥ 70 years old	164 (31.7)	29 (30.2)	135 (32.1)	0.81
Mechanical ventilation	122 (23.6)	45 (46.9)	77 (18.3)	<0.0001
ICU-level care	253 (48.9)	73 (76.0)	180 (42.8)	<0.0001
Chronic lung disease	24 (4.60)	2 (2.1)	22 (5.2)	0.28
Rheumatologic diseases	15 (2.9)	1 (1.0)	14 (3.3)	0.33
Asthma	34 (6.6)	2 (2.1)	32 (7.6)	0.65
Recent surgery	76 (14.7)	19 (19.8)	57 (13.5)	0.15
Chronic renal insufficiency	23 (4.4)	5 (5.2)	18 (4.3)	0.78
Central venous catheter	137 (26.5)	45 (45.9)	92 (21.9)	<0.0001
Pharmacological VTE prophylaxis	395 (76.4)	91 (94.8)	304 (72.2)	<0.0001
Death during hospitalization	73 (14.1)	23 (24)	50 (11.9)	0.003
Hospital stay, mean (SD)	13.2 (17.7)	24.4 (26.5)	10.6 (13.8)	<0.0001
≥ 48 hours	401 (77.6)	92 (95.8)	309 (73.4)	<0.0001
≥ 7 days	288 (55.7)	67 (69.8)	221 (52.5)	0.002
Chest CT abnormalities				0.015
Absent	92 (17.8)	13 (13.5)	79 (18.8)	
Mild	84 (16.2)	10 (10.4)	74 (17.6)	
Moderate	183 (35.4)	31 (32.3)	152 (36.1)	
Severe	158 (30.6)	42 (43.8)	116 (27.6)	
Other risk factors for VTE				
Previous VTE events	21 (4.1)	7 (7.3)	14 (3.3)	0.87
Diabetes mellitus (DM)	109 (21.1)	21 (21.9)	88 (20.9)	0.89
Systemic arterial hypertension (SAH)	246 (47.6)	47 (49)	199 (47.3)	0.82
Coronary artery disease (CAD)	43 (8.3)	7 (7.2)	36 (8.6)	0.84
Known thrombophilia	5 (1.0)	2 (2.1)	3 (0.7)	0.23
Paralysis of lower extremity	2 (0.4)	0 (0)	2 (0.5)	1.0
Cancer	21 (4.1)	5 (5.2)	16 (3.8)	0.57
D-dimer level, mean (SD)	4,773 (8,804)	12,021 (15,473)	3,121 (5,132)	<0.0001
BMI ≥ 25	419 (81)	74 (77.1)	345 (81.9)	0.312
BMI ≥ 30	140 (27.1)	35 (36.5)	105 (24.9)	0.03
Smoker	7 (1.4)	1 (1.0)	6 (1.4)	1
Former smoker	58 (11.2)	12 (12.5)	46 (10.9)	0.72

ICU, intensive care unit; CT, computed tomography; VTE, venous thromboembolism; BMI, body mass index; SD, standard deviation.

Lung involvement due to COVID-19 on chest CT was classified as absent (0%), mild (1-25%), moderate (26-50%), and severe (>50%). Moderate alterations were observed in 35.4% of patients, while severe changes were found in 30.6%. A total of 253 patients (48.94%) required ICU or step-down unit care, and 23.6% required MV. The mean length of hospital stay was 13 days (±18), which was longer in patients with VTE (24.4 days) compared to those without VTE (10.6 days) (p < 0.0001).

Approximately three-quarters (73; 76.04%) of the VTE events were detected among ICU and step-down unit patients, while 23 (23.96%) were detected in general wards. The incidence of VTE was 28.9% among ICU patients and 8.7% in general wards. Among the VTE events, 49 were pulmonary embolism (PE), 49 were deep vein thrombosis (DVT), four were catheter-associated DVT, and eight were superficial vein thrombosis (SVT). Some patients experienced more than one VTE event. The mortality rate was 14.1%, which was higher among VTE patients compared to those without VTE (24% vs. 11.9%) (p = 0.003). 

The IMPROVE-DD score is shown in Table 2. Overall, 50.9% of the patients had an IMPROVE-DD score ≥ 4. The mean score for all patients was 3.5 (± 2.6), significantly higher among patients with VTE (4.7 ± 3) compared to those without VTE (3.3 ± 2.4) (p < 0.0001). The most frequent variables contributing to the IMPROVE-DD score, which were also significantly more common in patients with VTE, included immobilization for ≥ 7 days, ICU/step-down unit admission, and D-dimer levels ≥ twice the upper limit of normal (2xULN). Although age ≥ 60 years was a frequent variable, it did not differ significantly between patients with and without VTE.

**Table 2:** IMPROVE-DD score among hospitalized patients with COVID-19 submitted to VTE investigation.

Risk factors	Points	All N = 517 (%)	With VTE N = 96 (%)	Without VTE N = 421 (%)	p-value
Previous VTE events, n (%)	3	21 (4.1)	7 (7.3)	14 (3.3)	0.09
Thrombophilia, n (%)	2	5 (1.0)	2 (2.1)	3 (0.7)	0.23
Paralysis of lower extremity during hospitalization, n (%)	2	2 (0.4)	0 (0)	2 (0.5)	1.0
Cancer, n (%)	2	21 (4.1)	5 (5.2)	16 (3.8)	0.57
Immobilization for at least 7 days, n (%)	1	288 (55.7)	67 (69.8)	221 (52.5)	0.002
ICU stay, n (%)	1	253 (48.9)	73 (76)	180 (42.8)	<0.0001
Age ≥ 60 years old, n (%)	1	253 (48.9)	50 (52.1)	203 (48.2)	0.5
Max D-dimer ≥ 2x ULN, n (%)	2	386 (74.7)	90 (93.8)	296 (70.3)	<0.0001
Sum of points	(0 to 14)				
Mean (±)		3.5 (2.6)	4.7 (3)	3.3 (2.4)	<0.0001
Median (IQR)		4	4 (2)	3 (2)	
Score 0-1, n (%)		97 (18.8)	5 (5.2)	92 (21.9)	<0.0001
Score 2-3, n (%)		157 (30.4)	19 (19.8)	138 (32.8)	
Score ≥ 4, n (%)		263 (50.9)	72 (75)	191 (45.4)	

VTE, venous thromboembolism; ICU, intensive care unit; ULN, upper limit of normal; IQR, interquartile range; SD, standard deviation.

Regarding the prediction of VTE risk based on the IMPROVE-DD score, 94.8% (91) of the patients with VTE had a moderate-to-high-risk score; 75% (72) had a high risk, with a score ≥ 4; and only 5.2% (4 men and 1 woman) of patients with VTE had a low-risk score (< 2). Of these, three scored “0” and two scored “1” due to age ≥ 60 years and ICU/step-down unit admission. Significantly more patients with VTE than without VTE had an IMPROVE-DD score ≥ 4 (75% vs. 45.4%) (p < 0.0001).

**Figure 1:**
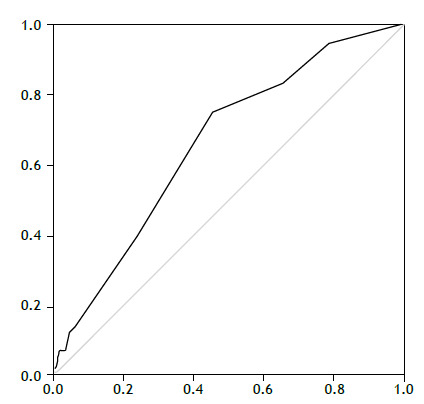
Receiver operating characteristic (ROC) curve for the IMPROVE-DD score and VTE. Notes (from top to bottom): first line, ROC curve; second line, null line.

The ROC AUC was 0.66 (Figure 1). In this sample of hospitalized patients with COVID-19 and suspected VTE, the sensitivity of IMPROVE-DD scores ≥ 4 for predicting in-hospital VTE was 75%, the specificity was 54.6%, the PPV was 27.4%, and the NPV was 90.5%.

## DISCUSSION

Among the COVID-19 patients evaluated for VTE in our study, 18.5% were found to have some form of VTE. A meta-analysis of 33 published trials conducted between January and June, 2020, involving 4,009 patients, reported an incidence of VTE in 9%, DVT in 3%, and PE in 8%. In ICU patients, these values increased to 21%, 8%, and 17%, respectively.^15^ In the present study, the incidence of VTE was three times higher in the ICU compared to general wards (28.9% vs. 8.7%), despite 94.8% of the patients who developed VTE receiving pharmacological VTE prophylaxis prior to diagnosis. A meta-analysis identified that the pooled incidence of VTE in studies where COVID-19 patients did not receive thromboprophylaxis was 21%, compared to 18.2% in studies where patients received standard-dose thromboprophylaxis, evidencing a minimal difference between the two groups.^6^ These findings are consistent with other systematic reviews and meta-analyses, highlighting the ongoing debate over the appropriate dose for effective prophylactic anticoagulation in these patients.^16^ ^,^ ^17^

Among the VTE events, PEs were more common than DVTs when compared to other clinically ill patient populations; however, most PEs in patients with COVID-19 were smaller in magnitude (segmental or subsegmental). On the other hand, patients with COVID-19 and VTE had significantly higher severity and hospital mortality rates (24% vs. 11.9%). This finding aligns with observations from other cohort studies.^18^ ^,^ ^19^

Significant risk factors for VTE included obesity, ICU/step-down unit admission, CVC use, prolonged hospital stays, more tomographic lung alterations/severity, the need for mechanical ventilation, and D-dimer levels at least 2xULN. As expected, patients with VTE had significantly higher D-dimer levels than those without VTE (mean: 12,021 vs. 3,121; p < 0.0001). Both the use of CVCs and elevated D-dimer levels are well-established risk factors for VTE and are incorporated into several risk assessment scores.^14^ ^,^ ^20^ ^-^ ^22^

Patients with VTE experienced worse clinical outcomes, including longer average lengths of stay in the ICU/step-down unit (24.4 vs. 10.6 days) and prolonged hospitalization. These outcomes are associated with case severity and have a direct relationship with thromboembolic events, primarily due to the promotion of venous stasis.^23^ ^,^ ^24^ Among the 73 deaths, 68 (93.2%) occurred in patients hospitalized for more than 7 days, and 71 (97.3%) were among those admitted to the ICU.

The need for mechanical ventilation, a clear indicator of severity, was identified as a risk factor for VTE: 46.9% of patients who received MV developed VTE, compared to 18.3% of those who did not. The mortality rate was significantly higher among COVID-19 patients requiring MV. This association has been consistently documented in the literature throughout the waves of the pandemic.^25^ ^-^ ^27^

Obesity is another factor associated with worse outcomes in COVID-19 and is also a known risk factor for VTE.^23^ ^,^ ^28^ ^,^ ^29^ The relationship between obesity, VTE, and COVID-19 is multifactorial and is believed to stem from chronic inflammation caused by obesity, which is exacerbated by COVID-19, as well as reduced mobility and endothelial injury resulting from the pro-inflammatory state.^23^ ^,^ ^30^ ^,^ ^31^ In the present study, 25% of patients had a BMI ≥ 30 kg/m^2^, and obesity was significantly more common among patients with VTE compared to those without VTE (36.5% vs. 24.9%, respectively).

Parenchymal changes observed on chest computed tomography, indicating more severe lung involvement due to COVID-19, were also predictors of poor outcomes, with a mortality rate of approximately one-quarter for patients with moderate findings and over half for those with severe findings. The use of imaging exams to predict severity and poor outcomes has been suggested in other studies and was supported by the findings of this sample.^32^ ^-^ ^34^

Other known risk factors for VTE and COVID-19 include active cancer, advanced age, lower limb paralysis, male sex, a previous history of VTE, known thrombophilia, smoking, diabetes, and cardiovascular diseases (*e.g*., SAH and CAD).^19^ ^,^ ^23^ ^,^ ^31^ ^,^ ^33^ ^-^ ^36^ However, in the studied sample, these factors were not significant in predicting VTE or poor outcomes.

A noteworthy finding of the present study was the ability to demonstrate, in a Brazilian cohort, that the IMPROVE-DD score can effectively identify the risk of in-hospital VTE in COVID-19 patients. The IMPROVE-DD score, proposed in 2017,^14^ has been evaluated in recent studies, including efforts to validate its use among COVID-19 patients.^8^ ^,^ ^10^ ^-^ ^12^ In a validation study involving 9,407 patients with COVID-19 between March and April 2020, researchers reported a sensitivity of 0.971, specificity of 0.218, PPV of 0.036, and NPV of 0.996. The AUC generated by the ROC curve was 0.703, and the IMPROVE-DD score showed a 6.8% better discrimination compared to the original IMPROVE score in COVID-19 patients, which was statistically significant.^8^ Similar findings have been reported by other authors.^9^

In the present study, IMPROVE-DD scores ≥ 4 were significantly associated with VTE, primarily driven by ICU/step-down unit admission, immobilization for ≥ 7 days, and D-dimer levels ≥ 2xULN. Notably, as a patient with a D-dimer level ≥ 2xULN scores “2” on the IMPROVE-DD scale, this single finding alone would be sufficient for the risk to be considered moderate. The MICHELLE study demonstrated improved combined clinical outcomes for VTE and mortality without an increase in major bleeding events by using extended post-hospital discharge prophylaxis with 10 mg/day rivaroxaban for 35 days, following in-hospital prophylaxis with 40 mg/day subcutaneous enoxaparin, in hospitalized COVID-19 patients with an IMPROVE-DD score ≥ 4, or a score of 2-3 with D-dimer levels > 500 ng/mL, provided they had a low risk of bleeding.^37^ Based on the IMPROVE-DD score cutoff of ≥ 4 to indicate the need for extended pharmacological VTE prophylaxis post-discharge, our findings suggest that 263 patients, or 50.9% of the total cohort, would be candidates for such prophylaxis, assuming no bleeding risk factors are present.

IMPROVE-DD criteria that were not significantly associated with VTE in our study included active cancer, lower limb paralysis, known thrombophilia, age ≥ 60 years, and a previous history of VTE. These risk factors were less common in our cohort, and the sample size was relatively small. However, age ≥ 60 years correlated with greater COVID-19 severity and higher mortality rates compared to the younger group (22% vs. 7%). The impact of aging, as highlighted in other studies,^8^ ^-^ ^10^ ^,^ ^19^ ^,^ ^25^ ^,^ ^27^ ^,^ ^33^ remains one of the most critical predictors of poor outcomes in severe COVID-19 patients. Although age did not show a significant association with in-hospital VTE in our cohort, it is an integral component of the IMPROVE-DD score.

An IMPROVE-DD score ≥ 4 does not serve as a diagnostic tool for in-hospital VTE due to its low sensitivity and specificity. However, it may be useful for ruling out in-hospital VTE in COVID-19 patients because of its relatively high negative predictive value (91%). In our study, 97 patients were categorized as “low VTE-risk” based on the IMPROVE-DD score. Nevertheless, five of these patients (5%) experienced thromboembolic events during hospitalization. Conversely, 81% of the patients (420/517) were classified as moderate-to-high VTE-risk and could be considered candidates for post-discharge VTE prophylaxis. This approach, however, could potentially result in the overuse of pharmacological prophylaxis. Prophylactic anticoagulation has potential drawbacks, including an increased risk of bleeding, high medication costs, drug interactions, and possible treatment non-adherence.^38^ ^,^ ^39^ Therefore, it is essential to carefully weigh the risks and benefits of post-discharge VTE prophylaxis.^40^ The original validation study of the IMPROVE-DD score reported a receiver operating characteristic (ROC) area under the curve (AUC) of 0.703.^8^ In the present study, the AUC ROC was 0.66, which is lower for in-hospital estimation. However, since the patients were not followed up after discharge, the ROC curve for the standard three-month follow-up period could not be calculated, representing a limitation of this study.

Finally, although many hospitalized COVID-19 patients have multiple risk factors for VTE and a high incidence of events despite receiving prophylactic anticoagulation with low-molecular-weight heparins during hospitalization, not all patients will benefit from extended post-discharge VTE prophylaxis. The use of IMPROVE-DD scores ≥ 4 in this population can aid in identifying higher VTE-risk patients; however, their risk of bleeding must be carefully assessed before they can be considered candidates for extended post-discharge VTE prophylaxis.
